# Three Generic Nevirapine-Based Antiretroviral Treatments in Chinese HIV/AIDS Patients: Multicentric Observation Cohort

**DOI:** 10.1371/journal.pone.0003918

**Published:** 2008-12-12

**Authors:** Taisheng Li, Yi Dai, Jiqiu Kuang, Jingmei Jiang, Yang Han, Zhifeng Qiu, Jing Xie, Lingyan Zuo, Yanling Li

**Affiliations:** 1 Department of Infectious Disease, Peking Union Medical College Hospital, Chinese Academy of Medical Sciences, Beijing, China; 2 Department of Epidemiology and Medical Statistics, School of Basic Medicine, Peking Union Medical College, Beijing, China; University of New South Wales, Australia

## Abstract

**Background:**

The purpose of this study was to evaluate the efficacy and safety of three nevirapine-based antiretroviral treatments for adult antiretroviral-naïve Chinese patients with HIV-1 infection.

**Methodology:**

This was a prospective, multicenter study. 198 antiretroviral-naïve HIV-1 positive subjects with CD4 lymphocyte counts between 100/ul and 350/ul and plasma HIV-1 RNA levels more than 500 copies/ml were randomized to start three NVP-based antiretroviral treatments: group A, NVP+AZT+ddI; group B, NVP+3TC+d4T; group C, NVP+AZT+3TC. Viral responses, immunologic responses, adverse events and drug resistence were monitored at baseline and the end of week 4, 12, 24, 36, 52. Viralogical response and immunological response were also comparaed in different strata of baseline CD4 T lymphocyte counts and plasma HIV-1 RNA concentrations. At baseline, the plasma HIV-1 RNA was 4.44±0.68, 4.52±0.71 and 4.41±0.63 lg copies/ml in group A, B and C respectively (p = 0.628). At the end of the study, the plasma viral load reached 2.54±1.11, 1.89±0.46 and 1.92±0.58 lg copies/ml in group A, B and C respectively (p<0.001). At week 52, suppression of plasma HIV-1 RNA to less than 50 copies/ml was achieved in more patients in group B and C than in group A (68.2%, 69% vs. 39.7%; p<0.001). In planned subgroup analyses, the decrease of viral response rate was seen in group A when CD4 cell count >200/ul (subgroup H). But in subgroup L, viral response rate of three groups has no significant statistic difference. There were no statistically significant differences among three groups in immunological response wthin any of the CD4 or pVL strata. 3 out of 193 patients with available genotype at baseline showed primary drug resistant. Of 26 patients with virologic failure, 17 patients showed secondary drug resistant, 16 subjects in group A and 1 subject in group B. Logistic regression analysis indicated that presence of hepatotoxicity was associated with HCV-Ab positive (OR = 2.096, 95%CI: 1.106–3.973, P = 0.023) and higher CD4 baseline (CD4 count >250/ul)(OR = 2.096, 95%CI: 1.07–4.107, P = 0.031).

**Conclusion:**

Our findings strongly support the use of 3TC+d4T and 3TC+AZT as the nucleoside analogue combination in NVP-based antiretroviral therapy. The regimen of AZT+ddI+NVP produced poor virological response especially in the stratum of CD4 count more than 200/ul. More patients showed secondary drug resistant in this arm too. Patients with HCV-Ab+ and CD4 count >250/ul appear to have significantly high risk of hepatoxicity.

**Trial Registration:**

ClinicalTrials.gov NCT00618176

## Introduction

Acquired immunodeficiency syndrome (AIDS) is a life-threating disease caused by human immunodeficiency virus (HIV). The advent of highly active antiretroviral therapy (HAART) has resulted in profound suppression of HIV replication, substantial increase of CD4+ T cells and partial reconstitution of the immune system.[1] All of these result in significant declines in morbidity and mortality from HIV/AIDS. In Western countries, twenty individual antiretroviral drugs, four fixed dose two-drug combinations, and two fixed dose three-drug combinations are available and many prospective, randomized clinical trials were performed to assess the efficacy and safety of HAART.[2] All these robust evidences were utilized to constitute the guidelines for occidental HIV/AIDS patients. In China, there are 650,000 people who are living with HIV infection, including about 75,000 AIDS patients.[3] Chinese government provide seven kinds of free-of-charge antiretroviral drugs which include Zidovudine (AZT), Stavudine (d4T), Lamivudine (3TC), Didanosine (ddI), Nevirapine (NVP), Efavirenz (EFV) and Indinavir (IDV). They are all generic drugs except for 3TC and EFV. About 16,000 AIDS patients have been treated with free-of-charge antiretroviral therapy. [4] The most popular drug combinations in China were NVP-containing regimens because of their convenience and tolerability. However limited data was come from trials of Chinese patients and no prospective randomized trial of different NVP-containing HAART strategies in treatment-naïve HIV-1 infected subjects was performed to give the evidences to verify their efficacy and safety in Chinese patients. To address this question, we compared three regimens containing the nonnucleoside reverse-transcriptase inhibitor NVP with the combination of two nucleoside reverse-transcriptase inhibitor AZT+ddI, 3TC+d4T or AZT+3TC with respect to antiretroviral potency, immunologic reconstitution, safety, tolerability, and the potential for drug resistence.

## Methods

The protocol for this trial and supporting consort checklist are available as supporting information; see Checklist S1 and Protocol S1.

### A. Patients

This study was approved by the ethics committee of Peking Union Medical College Hospital. Enrollment began in January 2005. Patient Information and Consent to Medical Treatment and sign a written consent form. Consenting individuals 18 years or older were recruited from thirteen research centers of China. They were found to be HIV-seropositive by standard serum enzyme-linked immunosorbent assay (ELISA) tests and also by Western blot analysis. Patients were considered for inclusion in this study if they were antiretroviral drug-naïve. The eligibility criteria for participants were CD4+ T-cell count from 100 to 350 cells/mm3; plasma viral load over 500copies/ml. Main exclusive criteria were pregnancy or breastfeeding, anticipated nonadherence, AIDS-defining illness within 2 weeks of entry, white blood cell count less than 2.0×109/L, absolute neutrophil count less than 1.0×109/L, hemoglobin level less than 90g/l, platelet count less than 0.75×1012/L, transaminase and alkaline phosphatase level more than 3 times the upper limit of the normal range, bilirubin level more than 2.5times the upper limit of the normal range, serum creatinine level more than 1.5 times the upper limit of the normal range.

Patients were randomly allocated to three treatment groups: group A, AZT(300mg twice daily [bd])+ddI (200mg bd or 125mg bd when less than 60kg)+NVP(200mg once daily for 2 weeks and 200mg bd thereafter); group B, 3TC(150mg bd)+d4T(30mg bd or 20mg bd when less than 60kg)+NVP; group C, AZT+3TC+NVP. (AZT, ddI, d4T and NVP are generic which made in China.) For farther analysis, patients were stratified by baseline CD4+T cell count: Subgroup L was defined as the baseline CD4+T cell ≤200/ul; subgroup H was defined as the baseline CD4+T cell >200/ul, and baseline plasma viral load (pVL): subgroup A, the pVL ≤10,000copies/ml; subgroup B, the pVL >10,000 and ≤100,000copies/ml; subgroup C, the pVL>100,000copies/ml. Patients were also stratified by baseline CD4+ T cell≤250/ul or CD4 + T cell>250/ul in the analysis of adverse effects.

### B. Methods

- The present study was divided into a screening period of 1–4 weeks and a follow-up period of one year. At the end of the screening period, the eligible patients were randomly allocated to one of the three treatment groups. The randomization was performed by drawing of envelopes. The follow-up period then started, with five visits at 4, 12, 24, 36, 52 weeks. During all of the visits the clinical assessment was recorded in the CRF and the subjects had samples taken for laboratory assessment and further detection including T cell subset, pVL and analysis of genotype resistance. The adverse events were classified and graded according to division of AIDS table for grading the severity of adult and pediatric adverse events.[5]
- Viral load:Plasma was separated from whole blood by centrifugation within 4h of collection and was stored frozen at −80°C until tested. The QUANTIPLEX HIV-1 RNA assay, version 3.0 (bDNA 3.0 assay), was performed according to the manufacturer's instructions that were provided with the assay kit. The limits of detection of the assay, indicated by the manufacturer, was 50∼500,000 HIV-1 RNA copies/ml.
- Immunofluorescent surface staining and flow cytometric analysis:Peripheral blood mononuclear cells were obtained by separation from the centrifugation gradient. Subpopulations of CD3+, CD4+, and CD8+ cells were determined by three-color flow cytometry (Beckman-Coulter, USA) at baseline and at the end of week 4, 12, 24, 36, 52. The following groups of monoclonal antibodies were used: PEcy5-CD4/PE-CD8/FITC -CD3 (CD4+/CD8+T cell counts). All monoclonal antibodies were purchased from Beckman-Coulter and Immunotech, USA.
- Blood routines, liver functions, sero-amylase, HCV-Ab, and serum lipids were administered by the clinical laboratory department of each research centers.
- Genotype resistance: Viral RNA was extracted from the isolated viruses using a QIAamp Viral RNA Mini Kit (Qiagen Inc., Chatsworth, CA) according to the manufacturer's protocol, and stored at −70°C until use. Reverse transcript reaction was run to synthesis of cDNA. The 1100-bp pol gene fragment encompassing the complete protease gene and the first 220 codons of the RT gene of HIV-1 RNA were amplified by nested PCR strategy and purified using a QIAquick Gel Extraction Kit (Qiagen Inc.). The product was sequenced and the results were compared to the consensus B reference sequence using HIVdb software (Stanford HIV Drug Resistance Database, http://hivdb.stanford.edu) to detect drug-resistance mutations. The protocol of nest PCR and DNA sequencing were described elsewhere.[6] Some of HIV-1 analysis was performed with the ViroSeq system according to the manufacturer's instructions as the quality control and remedy for in-house testing.
- Sample size and statistical analysis:
  - Sample size was calculated according to the both of the following criteria: CD4+ cells were detected at least 30 percent increasing than the baseline value; and the average efficient rates in CD4+ increasing of three treatment groups at least reached to 70 percent. In order to detect such a difference (with 80% power and 95% confidence) we need about 60 patients in each treatment group. Allowing for 20% loss to follow up, a total sample of 220 was required.
  - All statistical analysis was performed using the SPSS 11.5 statistical package. In present study, we performed two separate and complementary analyses of these data: the analysis according to the actual treatment and the intention-to-treat analysis. For the analysis according to actual treatment, we included only patients for whom outcomes were measured at specified time points. The intention-to-treat analysis involved patients who underwent randomization, received at least one dose of study medication, and had at least one plasma HIV-1 RNA measurement after treatment began. Missing values were imputed for the analysis with the use of a last-observation-carried-forward method. When a continuous outcome variable is considered, we use analysis of covariance to control for baseline variables. The change between the baseline value and the follow-up value of a particular outcome is evaluated by Kruskal-Wallis test or Mann-Whitney U test. Using a chi-square test, we compared the rates of response among the treatment groups in terms of the percentage of patients with suppression of plasma HIV-1 RNA levels to less than 400 copies per milliliter as well as the percentage for whom it was less than 50 copies per milliliter. Multivariate logistic regression analyses were used to identify factors associated with the presence of certain outcome. For all tests, P<0.05 was considered to be statistically significant.

## Results

### A. Baseline characteristics

**Figure 1:**
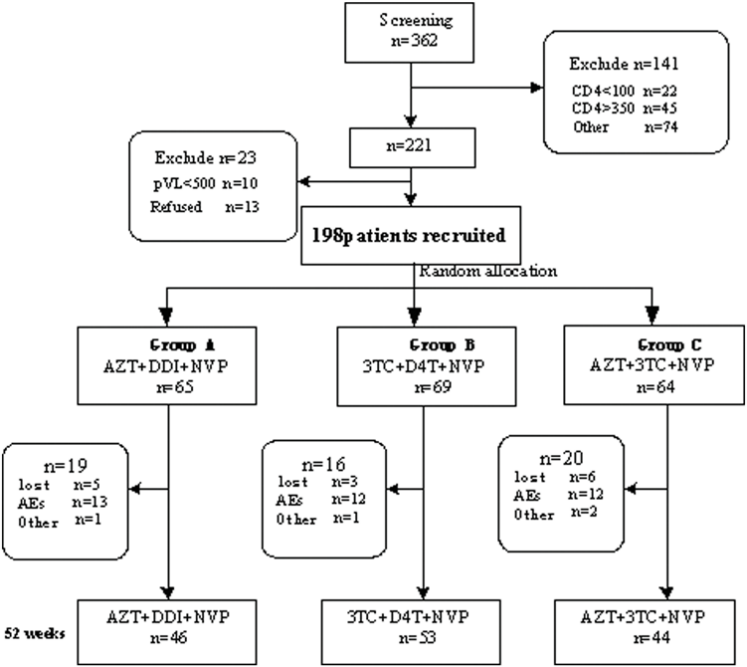
Disposition of patients (AZT, zidovudine; ddI, didanosine; NVP, nevirapine; 3TC, lamivudine; d4T, stavudine)

Of the 362 subjects screened, 198 HIV-positive antiretroviral-naive patients were recruited into this study. Demographic characteristics of the groups are summarized in Table 1. There were no statistically significant differences among the there treatment groups at baseline except for age, which was significantly older in the group B. The disposition of patients in the study shows in Figure 1.

**Table 1:** Demographic characteristics of patients of each group

	Intention-to-treat			Actual-treat		
	AZT+ddI+NVP Group A (n = 65)	3TC+d4T+NVP Group B (n = 69)	3TC+AZT+NVP Group C (n = 64)	AZT+ddI+NVP Group A (n = 46)	3TC+d4T+NVP Group B (n = 53)	3TC+AZT+NVP Group C (n = 44)
Male (n(%))	34 (52.3%)	31 (44.9%)	31 (48.4%)	24(52.2%)	23(43.4%)	24(54.5%)
Female (n(%))	31 (47.7%)	38 (55.1%)	33 (51.6%)	22(47.8%)	30(56.6%)	20(45.5%)
Age(years) Mean±SD	35.8±8.7	40.1±10.8	37.3±9.5	36.5(9.4%)	41.3(10.2%)	37.1(8.9%)
Prior AIDS (n(%))	2(3.1%)	2(2.9%)	4(6.3%)	0	2(3.8%)	2(4.5%)
CD4+ count(cells/ul) Mean±SD	221.8±73.4	218.5±84.0	224.0±73.5	234.2±71.0	207.8±75.0	219.6±70.8
Plasma HIV-RNA(log_10_copies/ml) Mean±SD	4.44±0.68	4.52±0.71	4.41±0.63	4.37±0.71	4.58±0.62	4.43±0.63
HCV-Ab positive (n(%))	21(32.3%)	21(30.4%)	16(25%)	15(34.1%)	14(32.6%)	9(23.1%)
HBsAg positive (n(%))	6(9.2%)	4(5.8%)	2(3.1%)	2(4.5%)	4(9.1%)	1(2.6%)

### B. Plasma viral load response

**Figure 2:**
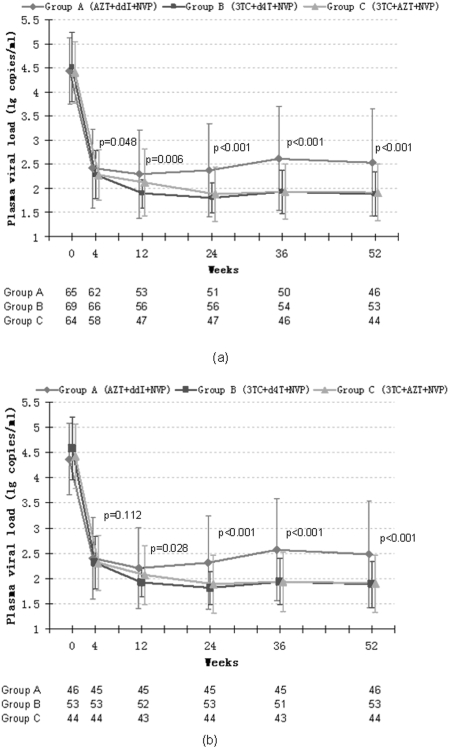
The plasma VL of three treatment groups. According to an analysis based on the intention-to-treat analysis (a) and actual treat analysis (b).P value is calculated by comparing the plasma VL of three groups.

In an intent-to-treat (ITT) analysis, the changes of plasma viral load during 52-week HAART in group A, B and C, are shown in Figure 2. After 4 weeks of HAART, plasma viral loads dropped by a mean of 2.14±0.57 lg copies/ml. At the end of the study, plasma viral loads dropped by a mean of 1.88±1.09 lg copies/ml in group A, 2.69±0.69 lg copies/ml in group B, 2.37±0.95 lg copies/ml in group C. The magnitude of the decrease in plasma VL of group B and C were significantly more than that of group A (P<0.001).

110 patients (58.8%) achieved a plasma VL of <50 copies/ml, 155 patients (82.9%) achieved a plasma VL of <400copies/ml. The percentage of patients who achieved a plasma VL of <50 copies/ml was 39.7% in group A, 68.2% in group B and 69% in group C (P<0.001). The percentage of patients who achieved a plasma VL of <400 copies/ml was 63.5% in group A, 92.4% in group B and 93.1% in group C (P<0.001).

**Figure 3:**
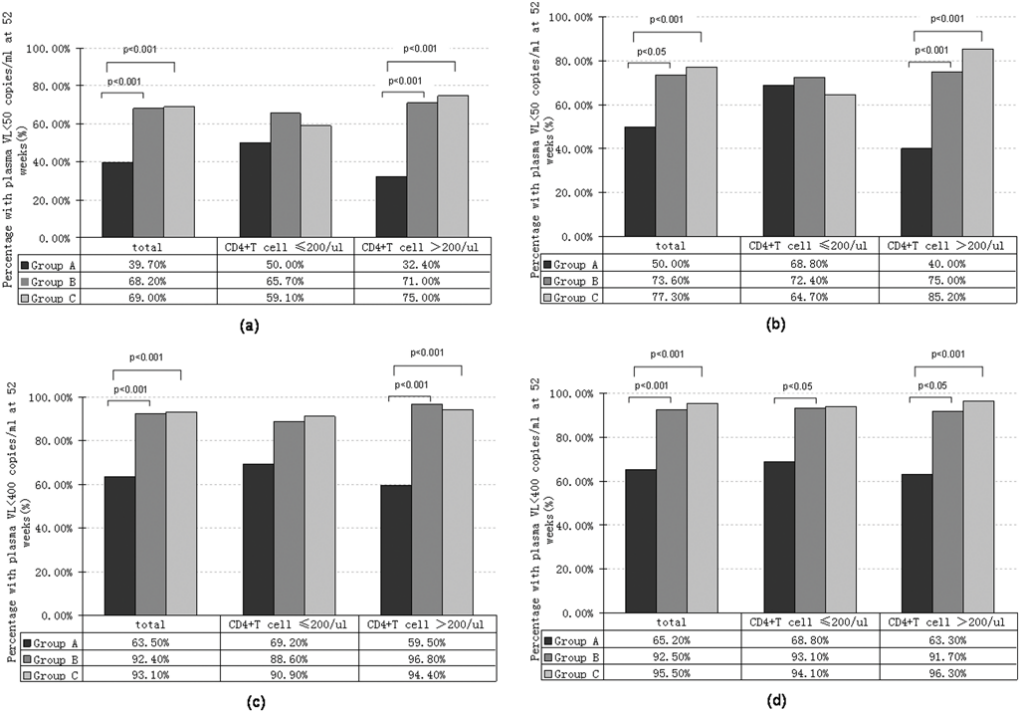
Percentage of patients with plasma VL of <50 copies/ml and <400 copies/ul at week 52. According to an analysis based on the intention-to-treat analysis (a, c) and actual tren\atment analysis (b, d). P values are calculated by pair wise comparing the viral response rate of two groups at week 52.

The viral response rate was analyzed when the subjects stratified by baseline CD4+T cell count. The baseline pVLs were 4.67±0.63 lg copies/ml in subgroup L and 4.29±0.66 lg copies/ml in subgroup H (P<0.001). At the end of 52 weeks, the pVL reached 2.07±0.80 lg copies/ml in subgroup L and 2.09±0.77 lg copies/ml in subgroup H (P = 0.744). The viral response rate of patients who divided into three therapy groups and stratified by baseline CD4+T cell count are showed in Figure 3. The viral response rate was higher in group B and C when the baseline CD4+T cell count was more than 200/ul (subgroup H). But this trend was not significant in subgroup L. When the subjects were stratified by baseline viral load, the viral response rates were 68.9%, 56.1%, 54.5% in subgroup a, b and c respectively (P = 0.285).

### C. Immunological response

**Figure 4:**
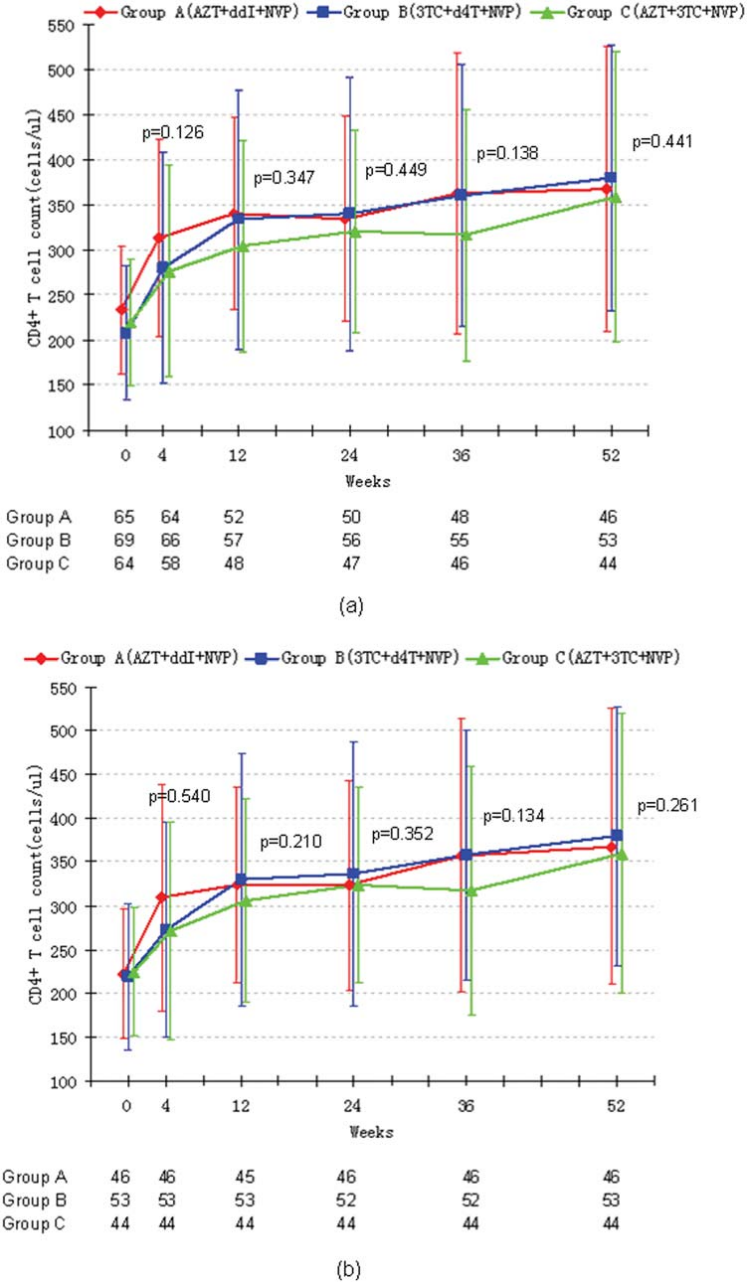
The increase of mean CD4+T cell count in three therapy group during HAART. According to an analysis based on the intention-to-treat analysis (a) and actual treat analysis (b). P value is calculated by comparing the CD4+ T cell count of three groups.

The increase in CD4+T cell count was divided into two phases: a rapid increase in the first 12 weeks constituted the first phase and a slow increase after week 12 constituted the second phase. The increased magnitudes of CD4+T cell count were 72/ul at week 4 and 105/ul at week 12. At the end of the study, the increased magnitudes of CD4+T cell count reached 149/ul. There was no significant difference among the mean CD4+T cell count of three groups at weeks 4, 12, 24, 36, 52. (Figure 4)

When the subjects stratified by baseline CD4+T cell count, the CD4+T cell count reached 289±128/ul at subgroup L and 432±144/ul at subgroup H (P = 0.581) at week 52. The increase trends of CD4+T cell count in two subgroups were parallel to each other. In each subgroup, there is no statistic difference of mean CD4+T cell count and the increased magnitute of CD4+T cell count in three treatment groups.

When the subjects stratified by baseline pVL, the baseline CD4+T cell counts were 250±68/ul in subgroup A, 216±74/ul in subgroup B, 195±73/ul in subgroup C (P<0.001). At the end of this study, CD4+T cell count reached 366±139/ul, 368±158/ul, 338±163/ul in subgroupA,B, C (P = 0.172).

There is no statistical difference of CD8+T cell count and CD4/CD8 ratio of three therapy groups at the baseline and at weeks 4, 12, 24, 36, 52.

### D. Clinical outcomes and adverse events

After 52 weeks of HAART, totally 14 patients (7.1%) lost to follow-up. In remain 184 patients, common complaints, such as fatigue, malaise and weight loss, were released at the end of this study. No one developed new AIDS-defining illness and died.

285 cases of adverse events occurred in 198 subjects. The details can be seen in Table 2. More than 85% adverse events mainly occurred in the early 12 weeks of the study except hyperlipidemia. The total AEs of each group has no statistic significant difference.

**Table 2:** The adverse events happened in this study in each treatment group (cases)

Adverse events	Treatment Groups									Possible corresponding drugs
	Group A			Group B			Group C			
	AZT+ddI+NVP			3TC+d4T+NVP			AZT+3TC+NVP			
	grade 2	grade 3	grade 4	grade 2	grade 3	grade 4	grade 2	grade 3	grade 4	
Hepatotoxicity	13	8	4	14	11	8	9	6	4	NVP
Hyperlipidemia	15	2	0	17	8	0	11	2	0	d4T/AZT/3TC
Neutropenia	5	3	0	7	2	1	8	6	4	AZT/3TC
Anemia	0	0	2	1	2	0	5	0	2	AZT/3TC
Thrombocytopenia	4	2	0	8	1	0	5	0	0	AZT/3TC
Rash	7	0	0	11	0	0	8	0	0	NVP
Gastrointestinal disorders	18	5	0	10	0	0	18	3	0	AZT/ddI
Peripheralneuropathy	2	0	0	1	1	0	1	0	0	ddI/d4T
Total	64	20	6	69	25	9	65	17	10	-

37 patients changed the corresponding drug because the adverse events were intolerable and could not be controlled by symptomatic treatment. (Figure 1) Bone marrow suppression mainly occurred in group C, in which the treatment contains AZT and 3TC.

In patients in all three groups, the most common treatment-related adverse events were hepatotoxicity. It occupied 47.1% severe adverse events (grade 3–4). The ratio of severe hepatotoxicity was 12/64(18.8%), 19/69(27.5%), 10/65(15.4%) respectively in treatment group A, B, C. Multivariate logistic regression analyses were used to identify factors associated with the presence of hepatotoxicity (grade 2–4). Patients have higher CD4 baseline (CD4 count >250/ul)(OR = 2.096, 95%CI: 1.07–4.107, P = 0.031) and HCV-Ab positive (OR = 2.096, 95%CI: 1.106–3.973, P = 0.023)were more likely to have hepatotoxicity after controlling for treatment group and the gender. If the subjects divided by the gender, logistic regression analyses showed that higher CD4 baseline (CD4 count >250/ul) increased the odds of developing hepatotoxocity in women (OR = 2.784, 95%CI: 1.128–6.874, P = 0.026) but not in men(P = 0.511). The bone morrow suppression occupied 29.9% severe adverse events (grade 3–4).

### E. Genotypic Analysis

Genotypic testing was carried out on baseline specimens and on specimens with pVL more than 1000copies/ml after 12 weeks. At baseline, among the 193 patients for whom HIV-1 RNA could be amplified, 3 subjects had isolates showing resistance mutations.

At virologic failure after 12-week therapy, of 26 patients without preexisting resistance, 17 patients had genotypic results available. Of the 17 with genotypic information available, 16 patients were in group A, and the other one was in group B. The mutations mostly occurred at 24-week therapy. Mutations related to NNRTI (K103N/R, Y181Y/C, G190A, V106A, V179T/D, Y188L/C, K101E, F227L) were detected in 17 patients. Mutations related to NRTI (M41L, A62V, K65R, D67N/S, T69N/S, K70R, L74V, V75M, V118I, M184V, L210W, T215Y, 151 Complex) were detected in 8 out of 17 patients.

### F. HCV coinfection

175 patients had HCV-Ab test at baseline. 58 subjects (29.3%) were HCV-Ab positive. The baseline HIV pVL of HCV(+) subjects and HCV(−) subjects were 4.43±0.72 copies/ml, 4.47±0.64 copies/ml (p = 0.75). The baseline CD4+T cell count of HCV(+) subjects and HCV(−) subjects were 231.1±75.6/ul, 218.0±75.3/ul (p = 0.281). At the end of this study, 57.4% HCV(+) patients and 57.9% HCV(−) patients achieved a pVL of less than 50copies/ml (p = 0.952). The increased magnitute of CD4+T cell count was 169.3±123/ul in HCV(+), 131.7±121/ul in HCV(−) (p = 0.162).

As we metioned before, logistic regression analysis indicated that presence of hepatotoxicity was independently associated with HCV-Ab positive (OR = 2.096, 95%CI: 1.106–3.973, P = 0.023).

## Discussion

NVP-based antiretroviral treatments are most accessible regimens in China. This was a prospective, multicentre study to evaluate three NVP-based regimens in terms of antiviral efficacy and tolerability in Chinese HIV-1 infected patients.

To date, information on the direct comparison among NVP-containing antiretroviral therapy has been scarce. The dissimilar criteria used of inclusion and exclusion in the studies makes it difficult to compare the efficacy of the regimens with different nucleoside backbones described in the different trial. In this study, we found significant difference over 52 weeks among three NVP-based regimens in the initial treatment of HIV-1 infection with regard to virologic response and resistance mutation. The regimen of AZT+ddI+NVP produced poor virological response especially in the stratum of CD4 count more than 200/ul. In the INCAS trail, the maximal effect in the AZT+ddI+NVP group was observed at week 16, with 68% of patients demonstrating a pVL<20 copies/ml, with 51% maintaining this level of suppression at week 52.[7] One of the inclusion criteria of the INCAS trial was CD4+T cell count between 200–600/ul. The discrepancy of virological response probably attribute to different ethnic group and CD4+T cell stratum. Few studies took the AZT+ddI+NVP regimen as an independent arm. So the result of this trial must be confirmed in other studies. Contrarily, the effect of treatment arm on the increase in CD4 count over time did not reach statistical significance.

In this study, two of the NVP-containing regimens: NVP+d4T+3TC, NVP+AZT+3TC, are effective in ART-naïve patients, producing a durable virological and immunological response. There are no significant differences over 52 week between these two regimens with regard to virological response, immunological response, adverse events or resistance mutations at virologic failure. In a comparison with an Australian trial of the same NRTIs with NVP, the 3TC+d4T and 3TC+AZT arm of the current study had similar proportion of patients at the end of the study with plasma HIV RNA <50copies/ml. [8] And the finding of comparable virological efficacy between these two regimens in suppression to a pVL<400 copies/ml is compatible with the results of a systematic review of triple combination therapies in antiretroviral therapy-naïve patients.[9] In this review, for patients using two NRTI and one NNRTI the overall percentage of patients achieving a pVL<400copies/ml after 48 weeks were 73% and the overall percentage of patients achieving a pVL<50copies/ml after 48 weeks were 64%. Our findings strongly support the use of 3TC+d4T and 3TC+AZT as the nucleoside analogue combination.

There were no statistically significant differences of virological response in any pVL or CD4 count strata. After 4-week therapy, there were no statistically significant differences of pVLs in any strata at all. And this trend lasted until the end of the study. Lower CD4 cell counts and higher viral loads at baseline were not associated with poorer virological outcome of HAART. A report from Andrew Phillips,[9] which was based on a large Europe cohort of patients starting HAART, found results broadly consistent with our own. On the other hand, in terms of immunological response, higher viral loads at baseline indicated greater increase in CD4 cell count. This is in line with one of the findings of the ACTG study.[10] This may be because of eliminating the high stress of viral load result in significant CD4 cell count recovery. But at the end of this study, the advantage of great increase in CD4 cell count disappeared gradually. In western country, large cohort study demonstrated disease progression to death and AIDS or death was clustered among patients starting antiretroviral therapy with high pVL.[11] It is needed to evaluate not only immunological response but also survival rate in a long-term assessment.

Primary resistance is common among adults recently infected with HIV-1 in North America and Europe. [12]–[14] But in this study, primary resistance rate is much lower than reports from western country. This discrepancy occurs because antiretroviral therapeutics was available in china much later than weastern country and many of Chinese AIDS patients have been treated with free-of-charge HAART since 2003. One of the main results of this study was that there was a trend toward higher drug resistance rate in AZT+ddI+NVP group. This result explained, at least in part, the higher rate of virologic failure in this group. The higher rate of gastrointestinal disorders and food restrictions of ddI in this therapy group may affect treatment comp1iance. This may be the intrinsic reason of virological failure and drug resistance.[15]

The safety profiles of the three therapeutic arms were also assessed in present study. Hepatotoxicity was the major adverse events of NVP-based therapy. Stern JO and his colleagues reported the increased risk of severe hepatotoxicity in women with CD4 count >250/ul and men with CD4 count >400/ul.[16] The result of this study is in line with the findings of Stern JO. The odds of hepatotoxicity increased significantly when baseline CD4 count is over 250/ul in all subjects and subgroup of women. The enrolling criterion of baseline CD4 count limits the analysis of odds of hepatotoxicity in men. This result supports the hypothesis that one of the mechanisms of hepatotoxicity is the hypersensitivity reaction.[17] To our knowledge this is the first study that reports the association between the incidence of hepatotoxicity and the stratified baseline CD4 count in Chinese.Another important mechanism of hepatotoxicity is chronic viral hepatitis coinfection. We also found the association between the occurrence of hepatotoxicity and HCV antibody positive. This result is in line with the report of Sulkowski et al.[18] The overall incidence of hepatotoxicity is 20.7%, a little higher than the reports of Sulkowski et al[18] and 2NN study.[19] This differences may be explained by demographics.

The incidence of hyperlipidemia in treatment group A and C is accordant with the result of 2NN.[19] A higher rate of hyperlipidemia was found in treatment group B in the present study. This is probably related to the presence of d4T in the backbone nucleoside combination, a NRTI that is more likely to cause hyperlipidemia than AZT and 3TC.[20] [21] But the lipodystrophy was not occurred in this study because of the relative short period of study.

All of the three therapeutic arms contain at lest one of AZT and 3TC. As the papers reported [22] [23], the hematologic toxic effects developed in three groups almost evenly. This adverse event occupied 29.9% severe adverse events of this study and mostly occurred in the early three months of the treatment.

The adverse events such as rash, gastrointestinal disorders, peripheralneuropathy are always mild and can be controlled by symptomatic therapy. But it is important to well handle these adverse events because it may affect the adherence of HAART.

In conclusion, as virological response and incidence of viral resistance has been concerned, 3TC+d4T and 3TC+AZT as the nucleoside analogue combination of NVP-based HAART is recommended. The diversity of immunological response of three NVP-based arm do not reach the statistic significant. We confirmed that incidence of hepatotoxicity is significantly higher in subgroup of CD4 count >250/ul and subjects with HCV-Ab positive in Chinese.

## Supporting Information

Checklist S1 CONSORT Checklist
(0.05 MB PDF)

Protocol S1 Trial Protocol
(0.07 MB PDF)
