# Supplementary material for: Three Generic Nevirapine-Based Antiretroviral Treatments in Chinese HIV/AIDS Patients: Multicentric Observation Cohort
Source: PLoS One. 2008 Dec 12;3(12):e3918. doi: 10.1371/journal.pone.0003918 (PMC2597744; doi:10.1371/journal.pone.0003918)
Supplement: Protocol S1 — Trial Protocol (0.07 MB PDF) [file pone.0003918.s002.pdf]

Protocol Registration Receipt
02/15/2008

Three Generic Nevirapine-Based Antiretroviral Treatments in Chinese Patients:Multicentric Observation Cohort

This study has been completed.

Sponsored by:	Peking Union Medical College<br>Ministry of Science and Technology of the People´s Republic of China
Information provided by:	Peking Union Medical College
ClinicalTrials.gov Identifier:	NCT00618176

Purpose

The purpose of this study is to determine whether the three generic nevirapine-based antiretroviral regimens are effective in the treatment of Acquired immune deficiency syndrome .

Condition	Intervention	Phase
Acquired Immunodeficiency Syndrome	Drug: Zidovudine (AZT)+ Didanosine (ddI)+ Nevirapine (NVP)<br>Drug: Stavudine (d4T), Lamivudine (3TC), Nevirapine (NVP)<br>Drug: Zidovudine (AZT), Lamivudine (3TC), Nevirapine (NVP)	Phase 4

Study Type: Interventional
Study Design: Treatment, Parallel Assignment, Open Label, Randomized

Further study details as provided by Peking Union Medical College:

Enrollment: 198

Study Start Date: January 2005
Primary Completion Date: September 2006

Arms	Assigned Interventions
Experimental: B	Drug: Stavudine (d4T), Lamivudine (3TC), Nevirapine (NVP)<br>Stavudine (d4T) 30mg bid (W>60Kg)20mg bid (W<60Kg) Lamivudine (3TC)300mg qd Nevirapine (NVP)200mg bid
Experimental: A	Drug: Zidovudine (AZT)+ Didanosine (ddI)+ Nevirapine (NVP)<br>Zidovudine (AZT) 300mg bid Didanosine (ddI) 200mg bid (W>60Kg)125mg bid (W<60Kg) Nevirapine (NVP) 200mg bid
Experimental: C	Drug: Zidovudine (AZT), Lamivudine (3TC), Nevirapine (NVP)<br>Zidovudine (AZT) 300mg bid Lamivudine (3TC) 300mg qd Nevirapine (NVP) 200mg bid

Eligibility

Ages Eligible for Study: 18 Years and older
Genders Eligible for Study: Both

Inclusion Criteria:

- 18 years or older
- the subjects were HIV-seropositive by standard serum enzyme-linked immunosorbent assay (ELISA) tests and also by Western blot analysis
- the subjects were antiretroviral drug-naïve
- a baseline CD4+ T-cell count from 100 to 350 cells/mm3 and a baseline plasma viral load over 500copies/ml

Exclusion Criteria:

- pregnancy or breastfeeding
- anticipated nonadherence
- AIDS-defining illness within 2 weeks of entry
- white blood cell count less than 2.0×109/L, absolute neutrophil count less than 1.0×109/L, hemoglobin level less than 90g/l, platelet count less than 0.75×1012/L
- transaminase and alkaline phosphatase level more than 3 times the upper limit of the normal range, bilirubin level more than 2.5times the upper limit of the normal range, serum creatinine level more than 1.5 times the upper limit of the normal range

Contacts and Locations

Locations

China

Peking Union Medical College Hospital

Beijing, China, 100730

More Information

Responsible Party:	Peking Union Medical College Hospital (Taisheng Li)
Study ID Numbers:	2004BA719A10, 2004BA719A10
Health Authority:	China:Ministry of Science and Technology
